# High rates of SARS-CoV-2 infection in funeral home workers in Ecuador: Is it an occupational risk for COVID-19?

**DOI:** 10.3389/fpubh.2022.1012434

**Published:** 2022-11-11

**Authors:** Esteban Ortiz-Prado, Jorge Eduardo Vásconez, Alexander Paolo Vallejo-Janeta, Diana Morales-Jadán, Aquiles R. Henriquez-Trujillo, Ismar A. Rivera-Olivero, Felipe Andrade, Tannya Lozada, Miguel Angel Garcia-Bereguiain, UDLA COVID-19 Team

**Affiliations:** ^1^One Health Research Group, Faculty of Health Sciences, Universidad de Las Américas, Quito, Ecuador; ^2^Universidad Latina de Costa Rica, San Jose, Costa Rica; ^3^Decanato de Investigación y Vinculación, Universidad de Las Américas, Quito, Ecuador

**Keywords:** SARS-CoV-2, funeral homes, COVID-19, Ecuador, occupational exposure

## Abstract

**Aim:**

The COVID-19 outbreak has already caused more than 6.5 million deaths, overwhelming health systems worldwide. The unusual demand for funeral home services could make these workers a potential risk group for occupational exposure to SARS-CoV-2 associated with corpses management for COVID-19 patients.

**Methodology:**

This is a cross-sectional study aimed to describe the infection rate of SARS-CoV-2 in funeral home staff by testing them with RT-qPCR in Quito, Ecuador. A total of 232 funeral home workers, representing more than 40% of funeral home personnel in Quito, were included in the study, in June 2020, immediately after the population lockdown was lifted in Ecuador.

**Results:**

A total of 48 individuals tested positive for SARS-CoV-2, yielding an infection rate of 20.7%. The SARS-CoV-2 infection rate was 18.1 and 20.0% among personnel managing corpses or not managing corpses, respectively. Among the SARS-CoV-2 positive patients, 81.3% reported no symptoms related to COVID-19, and 3 individuals had high viral loads over 10^8^ copies/ml.

**Conclusion:**

The high SARS-CoV-2 infection rate in funeral home staff suggested a potential occupational risk for COVID-19 but not related to corpses management. Public health guidelines for safe corpses management for COVID-19 victims and safe funeral services should be reinforced.

## Introduction

The COVID-19 pandemic has become the most severe public health problem of this century (1). Since its emergence, more than 650 million cases and more than 6.5 million deaths have been attributed to COVID-19 worldwide; nevertheless, the true pandemic death toll is currently estimated to be at least 22 million and not the official reports, due to a marked variance in official vs. excess mortality between countries (2–4). Massive COVID-19 outbreaks during the first 2 years of the pandemic have overwhelmed public and private health systems worldwide, especially in Latin-American regions (5–7). Overstretched intensive care units (ICUs), emergency departments, and hospitals were accompanied by dying unattended patients and corpses without proper burials (5–8).

The rapid emergence of new cases and the unprecedented death rate were so extreme that funeral services providers and cemeteries were overwhelmed, especially during the first wave of the COVID-19 pandemic (9, 10). This high demand for their services has only been previously seen during natural disasters such as earthquakes and or tsunamis (11, 12). This unusual demand and the uncertainty of managing potentially infectious dead bodies have established an occupational hazard for many front-line jobs, including those in charge of managing corpses from COVID-19 victims (13).

Since the very beginning of the pandemic, mortuaries and funeral homes have been maintaining thousands of dead bodies. This activity was suggested as a potential occupational risk of infection among funeral home workers through managing potentially infective bodies; the hazard that the COVID-19-related deceased could spread the SARS-CoV-2 virus linked to activities that could result in contact with bodily fluids including exposures to aerosols and the contact of fomites, but also contaminated surfaces (9, 14–17).

Moreover, mass gatherings during funeral services are also a potential source of infection for funeral home staff (9). Although there is no agreement on how the virus spreads from deceased patients, cautious handling of the remains is necessary given that the incidence of contamination of personal protective equipment following a complete autopsy ranges from 15 to 65% as reported by Mele et al. (17). To our knowledge, only one epidemiological report is available about the impact of SARS-CoV-2 among funeral home workers (10). Considering that Ecuador was one of the countries with the highest death toll per capita worldwide during the first year of the COVID-19 pandemic (13, 18), we aimed to describe the infection rate of SARS-CoV-2 among funeral home workers to assess a potential occupational risk associated with COVID-19.

## Methods

### Study design and setting

There are a total number of 19 funeral homes accredited to handle corpses that have died from COVID-19 in the city of Quito. According to the National Federation of Funeral Homes in Ecuador, at least 550 people are currently working in those funeral homes, including administrative personnel, funeral home support staff, mortuary transport technicians, funeral sales associates, funeral apprentices, and crematory technicians. A sample size calculation based on a margin error of < 5% and a confidence level of 95% assuming a response distribution of 50% among the respondents yielded a sample of 228 funeral home workers to achieve significance. A chain-referral sampling technique, which was deployed by the National Federation of Funeral Homes, was used to recruit workers.

A total of 232 workers (42.1% of the total population) were recruited and tested for SARS-CoV-2 and were included in this cross-sectional study to describe the incidence rate of SARS-CoV-2 infection among funeral homes staff in Quito, Ecuador, during June 2020. The socio-demographic information was obtained from the official epidemiological record used by the local health authority and the minister of public health (MoH).

### RNA extraction and RT-qPCR for SARS-CoV-2 detection

All samples were processed in the BSL2-certified molecular biology laboratory at Universidad de Las Americas, Quito (Ecuador). For nasopharyngeal sample collection, a rayon-tipped flexible swab was inserted into the naris until it reached the posterior nasopharynx. It was left in place for a few seconds and successively slowly withdrawn with a rotating motion. Swab tips were directly placed into 0.5 ml of TE of pH 8 buffer for SARS-CoV-2 diagnosis by RT-qPCR following an adapted version of the CDC protocol as we have previously described. Briefly, the CDC protocol is based on N1 and N2 probes to detect SARS-CoV-2 and RNase P as an RNA extraction quality control (19–27). Also, negative controls (TE pH 8 buffer) were included as a control for carryover contamination, one for each set of RNA extractions, to guarantee that only true positives were reported. For viral loads calculation, the 2019-nCoV N positive control (IDT, USA) was used, which was provided at 200.000 genome equivalents/μl, and a factor of 200 was applied to convert the viral loads to genome equivalents/ml and then converted to logarithmic scale (20–27).

### Statistical analysis

Measurements of frequency, central tendency, dispersion, and absolute differences were calculated for all categorical and continuous variables. A *t*-test analysis was used to compare parametric variables or a Wilcoxon–Mann–Whitney was used for non-parametric variables to asses differences when indicated. The statistic used for these contrasts is the difference in the means across all groups. We consider the confidence level at α = 0.01, α = 0.05, and α = 0.10.

## Results

### SARS-CoV-2 infection rates

**Figure 1:**
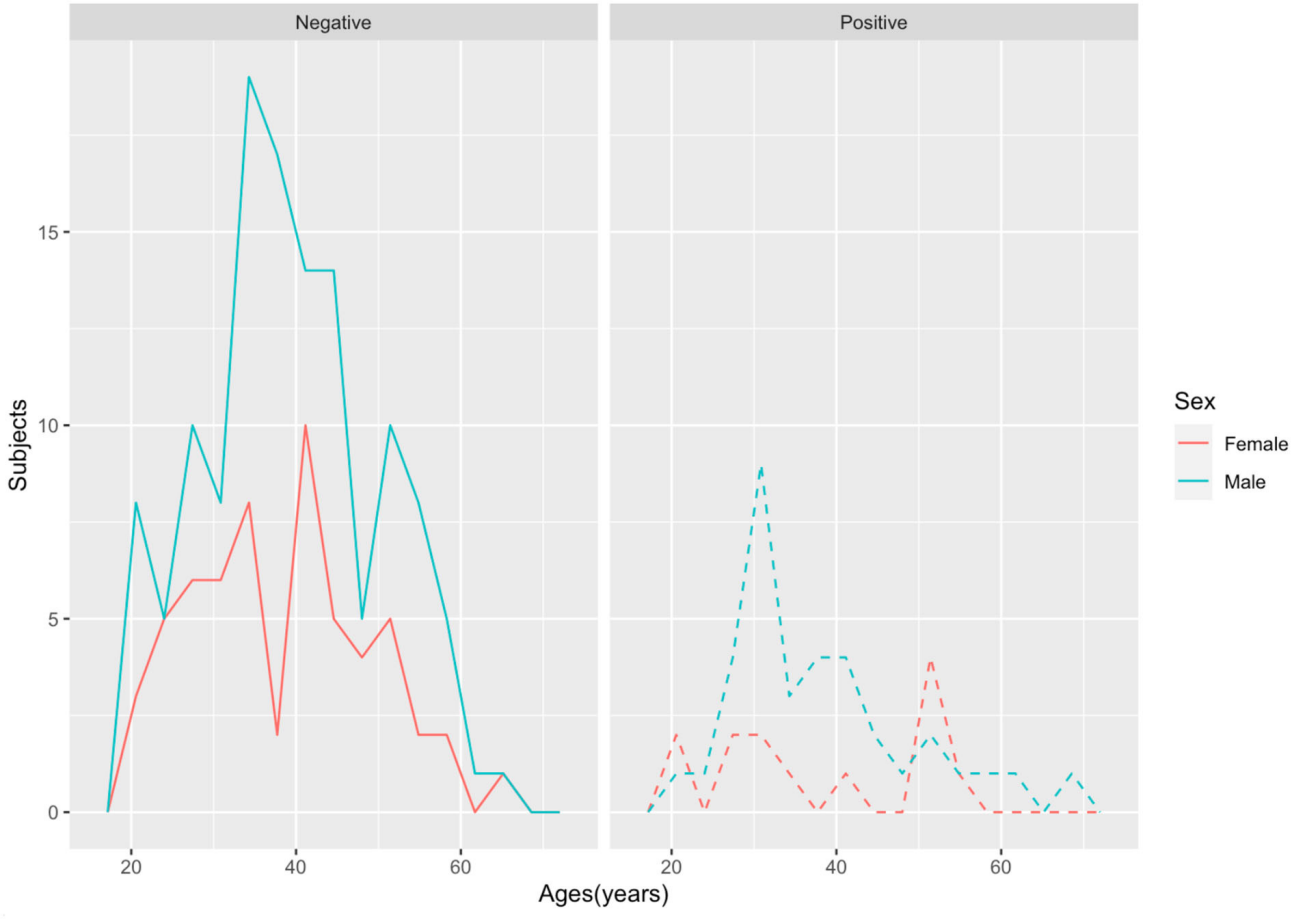
Age and sex distribution of SARS-CoV-2 test positivity among the 232 mortuaries' personnel.

A total of 48 out of 232 individuals tested positive for SARS-CoV-2, yielding a point prevalence of 20.7%. The number of positive cases in male staff was 35 out of 160 (21.9%), and the number of positive cases in female staff was 13 out of 72(18.1%), although those differences were not statistically significant. The average age for men and women was 38 and 39 years of age, respectively, and no statistically significant differences were found for SARS-CoV-2 positivity rates between age groups (Figure 1, Table 1).

**Table 1:** Sociodemographic and symptomatology status among COVID-19 funeral home workers.

			**COVID-19 results**			**Total**
			**Negative**	**Positive**	**Symptomatic**	
**Sex**	Male	*(n)*	125	35	2	160
		(%)	67,9%	72,9%	33,4%	69,0%
	Female	*(n)*	59	13	4	72
		(%)	32,1%	27,1%	66,6%	31,0%
Total		*(n)*	184	48	6	232
		(%)	100,0%	100,0%	100,0%	100,0%

Among the SARS-CoV-2 positive individuals, 39 (81.3%) reported no symptoms related to COVID-19, while 6 (12.5%) reported mild symptoms related to COVID-19 (3 out of 48 participants did not respond Table 1).

The SARS-CoV-2 infection rate was 18.1% for workers involved in corpse management and 20.0% for the staff not involved in corpse management, although those differences were not statistically significant.

### SARS-CoV-2 viral loads

**Figure 2:**
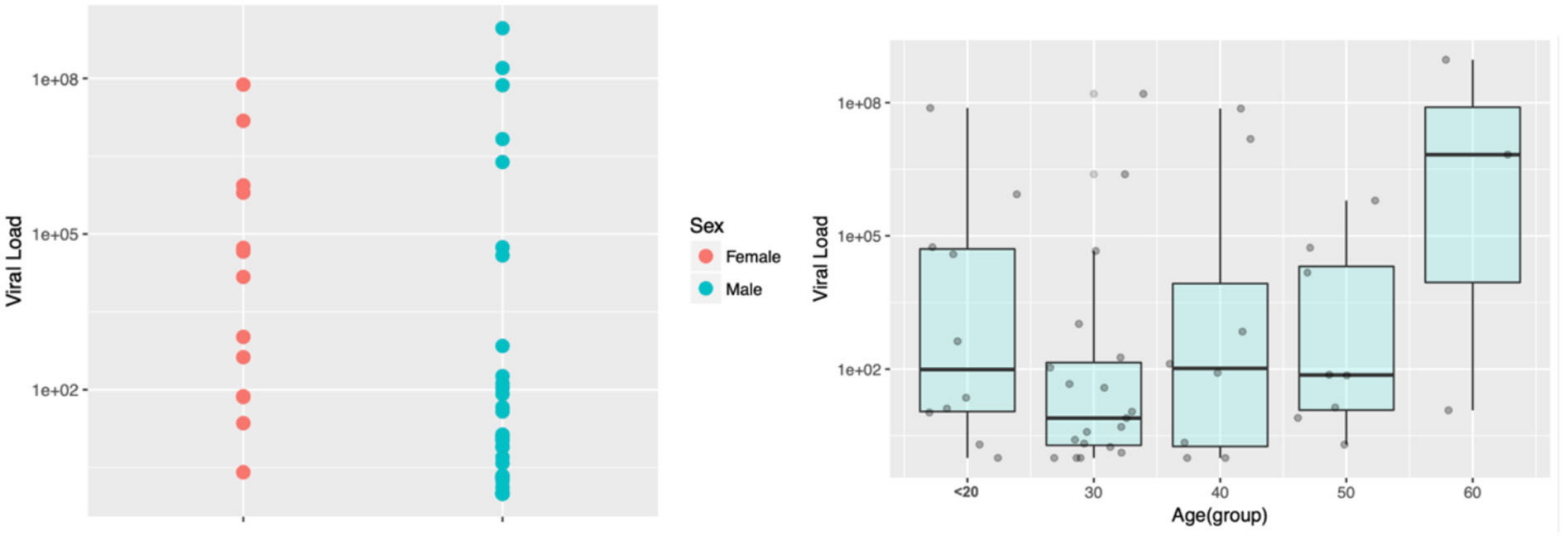
SARS-CoV-2 viral load distribution in the study population according to sex and age categories. Viral load is displayed in the log scale.

As it is illustrated in Figure 2, no statistical differences were found for the viral load neither between sex nor age categories. A total of 3 out of the 48 SARS-CoV-2 positive individuals (6.25%) had viral loads over 10^8^ copies/ml.

## Discussion

The number of COVID-19-related deaths has exceeded 6.5 million worldwide, although those numbers are far away from the total excess mortality reported during the COVID-19 pandemic (28, 29). So, the COVID-19 pandemic came along with massive mortality rates in short periods (30). For instance, in Ecuador, only 6 weeks after the first case of COVID-19 was confirmed by the end of February 2020, one of the most violent outbreaks in the world took place in the coastal region of the country; the provinces of Santa Elena and Guayas which usually report between 50 and 70 deaths per day, had more than 900 deaths per day during the first peak of the pandemic along March and April 2020, even overwhelming the funeral services (3, 13, 18, 31). As of December 31, 2020, Ecuador reported 9,473 confirmed deaths due to COVID-19 (32) and for the same date, an excess of 42,453 deaths was found in relation to the expected ones, thus, 32,980 could be the real excess deaths in this period, representing 348% of the deaths confirmed by COVID-19. This highest difference between excess deaths and the official COVID-19 deaths is similar to the data reported by Benitez et al. in which Ecuador for August 2020 presented 21,990 excess deaths representing 386% of the deaths reported by COVID-19 (33).

Under scenarios like the one described for Ecuador, funeral homes had to manage overloads of cases and even were obliged to keep the deceased in refrigerated trucks until it was possible to bury or cremate them. Moreover, corpses were kept at family homes for prolonged periods due to a shortage of capacity at funeral homes (13). When a person dies of COVID-19, the risk of transmission could depend on the viability of the virus in the body, on the surface of the body, or on fomites that may be contaminated with droplets or body fluids from the deceased (10, 32, 33). So, the mortuary staff have been potentially facing an occupational risk for SARS-CoV-2 infection associated with the management of the corpses to transport them to funeral homes and cemeteries (34, 35). The International Committee of the Red Cross described the risks associated with certain activities for personnel in contact with the deceased (34).

To our knowledge, this is the second epidemiological report addressing occupational risk for SARS-CoV-2 infection among funeral home staff. A recent study with mortuary workers from Qatar reported a high SARS-CoV-2 infection rate of up to 14.9% (10). Our study reported an even higher infection rate of up to 20.7% for SARS-CoV-2 in mortuaries personnel in the city of Quito in June 2020, a few weeks after the mandatory lockdown was lifted. Although occupational risk related to corpse management could not be concluded from our results, the high SARS-CoV-2 infection rate obtained among funeral home staff would suggest an occupational risk. For instance, these workers were on duty while most of the population was in lockdown, and they were in touch not only with corpses but also with family members of COVID-19 victims. Thus, either directly due to funeral home activity or indirectly due to community transmission among front-line workers during population lockdown, occupational risk cannot be ruled out. The infection rates were as high as for other front-line risk groups as healthcare workers or food delivery riders (36). Although a similar study carried out in Qatar suggested that community transmission is more than the occupational risk for mortuary workers (10), in the context of Ecuador, an infection rate of higher than 20 % is worrisome and could not be simply explained by community transmission. We pointed out that during the same period, the MoH reported a 40% prevalence of SARS-CoV-2 infection among hospitalized patients. Moreover, this study was carried out a few weeks after the lifting of the lockdown in the draconian population in June 2020 that, except for the province of Galapagos (37), was followed by COVID-19 outbreaks countrywide (5, 38–43). So, funeral home workers are clearly among the most vulnerable population for occupational risk for SARS-CoV-2 infection. Furthermore, most of the SARS-CoV-2 positives cases among funeral home workers were asymptomatic, increasing the risk of transmission to the community. Although we were advised that proper PPE should be provided to every funeral worker in Ecuador, during the initial peak of the pandemic even front-line health personnel had trouble finding proper PPE (44–46). Thus, poor PPE allocation to funeral home workers cannot be excluded.

This study has several limitations. The first is the lack of contact tracing information for the positive individuals due to either occupational or community transmission is an important limitation to clarify in the SARS-CoV-2 infection. The second is the reduced number of individuals that completed the occupational survey did not allow to have a stronger statistical analysis of the occupational risk associated with corpses management. Finally, the lack of information about PPE availability and proper use was also a limitation to be explained if the high attack rate of SARS-CoV-2 observed was related to occupational risk.

The high prevalence of SARS-CoV-2 infection found in funeral homes staff in Quito showed that this population is a vulnerable population due to occupational risk of infection, with a similar prevalence to healthcare workers, and as a potential source of transmission of SARS-CoV-2 to the community. Funeral homes should implement a frequent preventive SARS-CoV-2 testing scheme in their employees and develop proper safety protocols for their employees and customers.

## Data availability statement

The original contributions presented in the study are included in the article/supplementary material, further inquiries can be directed to the corresponding authors.

## Ethics statement

All participants signed informed consent to participate freely and voluntarily in this SARS-CoV-2 testing surveillance. This study is a secondary analysis of the anonymized laboratory results from previous surveillance testing carried out in the context of the COVID-19 pandemic. Nevertheless, the study was approved by the Institutional Review Board from Hospital General San Francisco (Quito) with code CEISH-HGSF-2021-002.

## UDLA COVID-team

Byron Freire-Paspuel, Tatiana Jaramillo, Daniela Santander Gordon, Gabriel Alfredo Iturralde, Julio Alejandro Teran, Karen Marcela Vasquez, Jonathan Dario Rondal, Genoveva Granda, Ana Cecilia Santamaria, Cynthia Lorena Pino, Oscar Lenin Espinosa, Angie Buitron, David Sanchez Grisales, Karina Beatriz Jimenez, Heberson Galvis, Barbara Coronel, Vanessa Bastidas, Dayana Marcela Aguilar, Ines Maria Paredes, Christian David Bilvao, Maria Belen Paredes-Espinosa, Sebastian Rodriguez Pazmiño, Juan Carlos Laglaguano, Henry Herrera, Pablo Marcelo Espinosa, Edison Andres Galarraga, Marlon Steven Zambrano-Mila, Ana Maria Tito, Nelson David Zapata.

## Author contributions

All authors listed have made a substantial, direct, and intellectual contribution to the work and approved it for publication.

## Funding

This study was funded by Universidad de Las Américas and by Fundación CRISFE (Fondo Sumar juntos).

## Conflict of interest

The authors declare that the research was conducted in the absence of any commercial or financial relationships that could be construed as a potential conflict of interest.

## Publisher's note

All claims expressed in this article are solely those of the authors and do not necessarily represent those of their affiliated organizations, or those of the publisher, the editors and the reviewers. Any product that may be evaluated in this article, or claim that may be made by its manufacturer, is not guaranteed or endorsed by the publisher.
